# Efficacy Evaluation of an E2 Subunit Vaccine Against Highly Virulent Classical Swine Fever Virus Strain

**DOI:** 10.3390/vaccines13101072

**Published:** 2025-10-20

**Authors:** Yu-Chieh Chen, Chi-Chih Chen, Wen-Bin Chung, Yen-Li Huang, Guan-Ming Ke, Hso-Chi Chaung

**Affiliations:** 1 International Degree Program in Animal Vaccine Technology, National Pingtung University of Science and Technology, Pingtung 912301, Taiwan; ycchen@mail.npust.edu.tw; 2 Research Center for Animal Biologics, National Pingtung University of Science and Technology, Pingtung 912301, Taiwan; chen0100@mail.npust.edu.tw (C.-C.C.); wbchung@mail.npust.edu.tw (W.-B.C.); 3 Department of Veterinary Medicine, National Pingtung University of Science and Technology, Pingtung 912301, Taiwan; 4 Department of Post-Baccalaureate Veterinary Medicine, Asia University, Taichung 413305, Taiwan; ellis7374365@asia.edu.tw; 5 Graduate Institute of Animal Vaccine Technology, College of Veterinary Medicine, National Pingtung University of Science and Technology, Pingtung 912301, Taiwan

**Keywords:** classical swine fever, subunit vaccine, CpG adjuvant, DIVA strategy

## Abstract

**Background/Objectives**: Classical swine fever (CSF) is listed by the World Organisation for Animal Health as a highly devastating and contagious pig disease, causing severe economic losses to the swine industry. In spite of the successful elimination of CSF in Taiwan, preparedness against potential reintroduction remains essential. The live attenuated vaccines have been effective in disease control, but are not capable of a viable strategy that differentiates infected from vaccinated animals (DIVA). Subunit vaccines are recognized for their safety and ability to induce protective immunity against infectious diseases. **Methods**: In this study, the recombinant CSF virus (CSFV) E2 proteins were formulated with a CpG motif as an adjuvant to produce the E2-CpG subunit vaccine. Its efficiency in specific-pathogen-free (SPF) pigs was compared with a commercially available E2 subunit vaccine (Bayovac^®^ CSF-E2; Bayer Taiwan Co., Ltd., Taipei City, Taiwan). **Results**: Significantly higher titers of anti-E2 antibodies were induced in pigs immunized with a single dose of the E2-CpG vaccine, particularly the reduced E-0.5A formulation, than those immunized with a dose of the commercialized E2 subunit vaccine adjusted to double dosage. This designed subunit vaccine showed high efficacy in protection against clinical symptoms and significant pathological alterations in pigs after a highly virulent CSFV (genotype 1.1) challenge. Viral shedding was not detected in vaccinated pigs before completion of the challenge study, and the viral load in their spleens remained undetectable. **Conclusions**: These results could support the potential of the E2-CpG vaccine as a cost-effective, single-dose subunit vaccine capable of inducing robust CSFV-specific immunity and providing 100% protection against lethal CSFV challenges.

## 1. Introduction

Classical swine fever (CSF) is a devastating and highly contagious pig disease caused by classical swine fever virus (CSFV). CSFV can infect both domestic pigs and wild boar. The mortality rate in infected animals may be as high as 100% in CSF-epidemic areas [1], although CSF affects the swine industry worldwide. The World Organisation for Animal Health (WOAH) lists CSF as an infectious animal disease that requires reporting immediately once the outbreak is found [2]. CSFV is genetically diverse and is currently classified into three major genotypes (1, 2, and 3) and multiple subgenotypes [3,4,5]. In East and Southeast Asia, genotype 2.1 strains, including highly virulent isolates such as the ALD strain (genotype 1.1), have been predominant in recent outbreaks, emphasizing the need for vaccines capable of providing cross-protection against diverse genotypes [6,7]. It is one of the most economically impactful diseases, especially in countries with a high density of raising pig populations.

Vaccination remains the best way to control this disease. The traditional live attenuated C-strain vaccine has been widely used for decades because it is both inexpensive and effective in preventing disease outbreaks [8]. Despite the outstanding effectiveness and safety of this vaccine, it is impossible to differentiate infected from vaccinated animals (DIVA). This is the main disadvantage of this vaccine, posing a major limitation for disease surveillance and eradication programs. When implementing effective disease control strategies in CSF-epidemic areas, the live attenuated vaccines caused complications in serological monitoring and made it difficult to confirm the absence of field virus circulation. Therefore, developing a safe and effective subunit vaccine compatible with DIVA diagnostic systems—such as those detecting antibodies against E^rns^ or NS3—is crucial for CSF eradication and long-term control. Hence, this study aimed to develop and evaluate an effective E2 subunit vaccine that could serve as a DIVA vaccine, providing both protective efficacy and practical utility for eliminating this highly contagious disease.

## 2. Materials and Methods

### 2.1. Expression and Purification of Recombinant CSFV E2 Antigen Subsection

The recombination CSFV E2 protein was expressed in the FlashBac™ expression system (Oxford Expression Technologies Ltd., Oxford, UK). The CSFV E2 glycoproteins (nucleotide positions 1201-2294) were derived from epidemic strain genotype 2.1a (AY526726) genes. To enhance E2 protein secretion and solubility, the modified sequence was constructed by adding a heterologous signal peptide, deleting the native transmembrane domain, and including a C-terminal 6× His-tag for purification. The sequence was synthesized by Genomics Co., Ltd. (New Taipei City, Taiwan), and the information is shown in Table S1. Briefly, the pBacPAK8-SPdTM was constructed and transformed into an *E. coli* expression system to produce the expression vector. The recombination baculovirus with pBacPAK8-SPdTM expression vector was used to infect High Five™ Cells (Cat. No. 3855-02; Thermo Fisher Scientific, Carlsbad, CA, USA) for E2 expression. The soluble CSFV E2 antigen was harvested and purified onto a Ni^+^-NTA column (HisTrap™ HP, Merck KGaA, Freiburg, Germany) as described by the manufacturer’s instructions. This expressed recombination CSFV E2 antigen was then verified by SDS-PAGE after purification. The molecular weight of the mainly expressed native purified recombinant E2 antigen was about 82 kDa. The monomer under the reducing reagent β-mercaptoethanol (β-ME) appeared to have a molecular weight of 41 kDa (Figure S1a). The purified recombinant E2 antigen was further confirmed by Western blot analysis using E2-specific monoclonal antibody (mAb) WH303 (APHA Scientific, Loughborough, UK) (Figure S1b). The final product of soluble E2 antigen was frozen in liquid nitrogen and stored at −80 °C.

### 2.2. Vaccine Preparation

The soluble E2 antigen was formulated with or without a porcine-specific CpG oligodeoxynucleotide (CpG-ODN) adjuvant. The CpG-ODN sequence is proprietary (United States Patent No. US 10117929 B1), and is a fully phosphorothioate-modified TLR9 agonist with multiple CpG motifs known to activate antigen-presenting cells and upregulate CD80/CD86 expression in porcine PBMCs. Therefore, these porcine-specific CpG-ODNs were used as vaccines in vaccination protocols for piglets. All vaccines were dissolved in a total volume of 2 mL of Montanide™ ISA206VG (SEPPIC, Puteaux, France), which is a water-in-oil emulsion.

### 2.3. Animal Trials

All specific-pathogen-free (SPF) piglets were obtained from the Animal Technology Research Institute (ATRI, Miaoli, Taiwan). Animal trial I was approved by the Institutional Animal Care and Use Committee (IACUC) of National Pingtung University of Science and Technology (NPUST) under compliance number 110-040 (5 July 2021 at approval). The CSFV challenge experiment in animal trial II was approved by the Veterinary Research Institute, Ministry of Agriculture (AHRI, Miaoli, Taiwan), under IACUC number 110-X03 (30 July 2021 at approval). All animals were provided ad libitum diets and housed in high-containment animal biosafety level II (ABSL-2) facilities.

The vaccination treatments for both animal trials are summarized in Table 1. In animal trial I, a total of 17 SPF pigs at the age of 4 weeks were randomly divided into 6 groups. All the pigs were primarily vaccinated intramuscularly at the age of 4 weeks. The pigs in Groups E-1, E-1A, and E-0.5A were only inoculated with a single dose of vaccine, while those in Group E-2 received a booster administration 2 weeks post-primary vaccination. The pigs in Group C were injected with a commercial CSFV E2 subunit vaccine (Bayovac^®^ CSF-E2; Bayer Taiwan Co., Ltd., Taipei City, Taiwan) that was adjusted to a single dose with 64 μg/dose of CSFV E2 glycoprotein in 2 mL. Those in Group A were inoculated with CpG adjuvant without E2 antigen only as a non-vaccinated control group. The serum of each animal in all groups was collected prior to vaccination and at 4 weeks after primary vaccination and stored at −80 °C for the subsequent assay.

In animal trial II, a total of nine SPF pigs at the age of 4 weeks were randomly divided into three groups for the challenge study. In accordance with the results of animal trial I, vaccine formulas of Groups E-1A and E-0.5A were chosen for the challenge study. The animals in Group N were administered with saline only as the control group. All the pigs were challenged intramuscularly with 2 mL of the CSFV ALD strain (10^5.6^ FAID_50_/mL; genotype 1.1) [9,10], adhering to the Animal Drugs Inspection Branch of AHRI’s guide, at the beginning of day 28 under BSL-3 conditions. The surviving animals were euthanized 14 days after CSFV challenge and samples were collected regularly under the WOAH Reference Laboratory Guidance. Daily clinical evaluations were performed for all pigs after CSFV challenge by veterinarians in addition to regular monitoring of body temperature and body weight. Sera, nasal, and oral swab samples were collected before vaccination and at 0, 3, 7, 10, and 14 days after CSFV challenge. Following the AVMA euthanasia guidelines (2020 edition), spleen samples were also collected at necropsy. RNA samples were extracted from sera, nasal swabs, oral swabs, and spleens of pigs and stored at −80 °C for subsequent assays. After euthanasia or death, necropsies were performed and pathological parameters were recorded.

### 2.4. Examinations of E2-CpG Vaccines Vaccination Effectiveness

All the pigs were monitored daily for clinical signs by veterinarians, and the body weights and rectal temperatures of the pigs were monitored regularly in both animal trials. The sera samples were collected to evaluate E2-specific antibody responses using the IDEXX CSFV Ab Test kit (IDEXX Laboratories, Bern, Switzerland) according to the manufacturer’s instructions. ELISA measures antibodies in the test sample capable of inhibiting the binding of CSFV E2-specific mAb, and the results are expressed as blocking percentages. Samples with blocking values ≥ 40% were considered positive for anti-E2 antibodies.

In animal trial II, RNA contents in the sera, nasal swabs, saliva swabs, and spleen were extracted with the PureLink^TM^ Viral RNA/DNA Mini Kit (Invitrogen^TM^, Carlsbad, CA, USA). In accordance with Hoffmann et al.’s (2005) [11] method with minor adjustments, quantitative detection of the viral RNA was performed using a one-step RT–PCR protocol. The set target is a 93 bp fragment of the 5′ untranslated region of CSFV. It was inspected using the QuantiTect^TM^ probe RT–PCR kit (Qiagen, Hilden, Germany) with a CSF-specific primer–probe: forward primer CSF100-F (5′-ATG CCC AYA GTA GGA CTA GCA-3′), reverse primer CSF192-R (5′-CTA YTG ACG ACT RTC CTG TAC-3′), and probe (5′-FAM-TGG CGA GCT CCC TGG GTG GTC TAA GT-TAMRA-3′) [11]. RT–PCR amplification was performed on a QuantStudio^TM^ 3 RT–PCR system instrument (Thermo Fisher Scientific, Singapore) with the following cycling conditions: 50 °C for 30 min (reverse transcription), 95 °C for 5 min (enzyme activation), followed by 40 cycles of 94 °C for 15 s (denaturation), 57 °C for 30 s (annealing), and 68 °C for 30 s (extension). The same primers were used as a positive and an internal control plasmid. The cycle threshold value (Ct) of each reaction was then calculated via QuantStudio™ Design & Analysis software version 1.5.1 (Invitrogen™, Palo Alto, CA, USA), and viral RNA copy numbers were quantified using a plasmid-derived standard curve (10^11^−10^0^ copies/mL). Results were expressed as Log copies/mL for fluids and Log copies/g for tissue samples.

The body temperature was recorded, and fever was defined as a rectal temperature higher than 40.5 °C according to the criteria described by Mittelholzer et al. (2000) [12]. Then clinical signs (loss of appetite, depression, shivering, hemorrhage, and diarrhea) of each animal were monitored daily from day 1 to day 14 after CSFV challenge. The clinical scores based on the method of Mittelholzer et al. (2000) were recorded by veterinarians during CSFV challenge [12]. In addition, pathological parameters were measured via a slightly modified version of the clinical score developed by Floegel-Niesmann et al. (2003) and Malswamkima (2015), including the scoring of gross lesions and histopathological lesions [13,14]. The gross lesions were scored on a 0 (no lesion) to 3 (severe CSF lesion) scale [13]. For histopathological examinations, lymphoid tissues and target organs were fixed in 10% neutral-buffered formalin solution, embedded in paraffin, and sectioned at 2 μm thickness before being stained with hematoxylin–eosin. The slides were examined under an optical microscope (Olympus BX51, Taiwan) at 40 to 600× magnification. According to the protocol of Malswamkima (2015), histopathology was scored on a 0- to 3-point scale (0 = normal, 1 = mild, 2 = moderate, 3 = severe) [14]. The two types of clinical lesions were examined for lesions associated with CSF in major tissues (cerebrum, spleen, kidney, lymph nodes, stomach, and ileocecal valve) and considered together. The maximum total pathology score possible was 18, which described all parameters being considered as severe CSF symptoms.

### 2.5. Statistical Analysis

All the data were compared, analyzed, and graphed with the SAS deployment wizard v9.4 (SAS institute Inc., Cary, NC, USA) and GraphPad Prism 5 (GraphPad Software, Boston, MA, USA). Differences among multiple experimental groups were evaluated by one-way analysis of variance (ANOVA) followed by Duncan’s multiple range test. Survival data were analyzed using the Mantel–Cox log-rank test. Data were expressed as the mean ± standard error of the mean (SEM). Statistical significance was defined as *p* < 0.05. All datasets were tested for normality (Shapiro–Wilk test) before applying parametric tests; when data did not meet normality assumptions, equivalent non-parametric tests (Kruskal–Wallis with Dunn’s post hoc) were applied.

## 3. Results

### 3.1. Development of E2-Specific Antibodies in Animal Trial I

**Figure 1:**
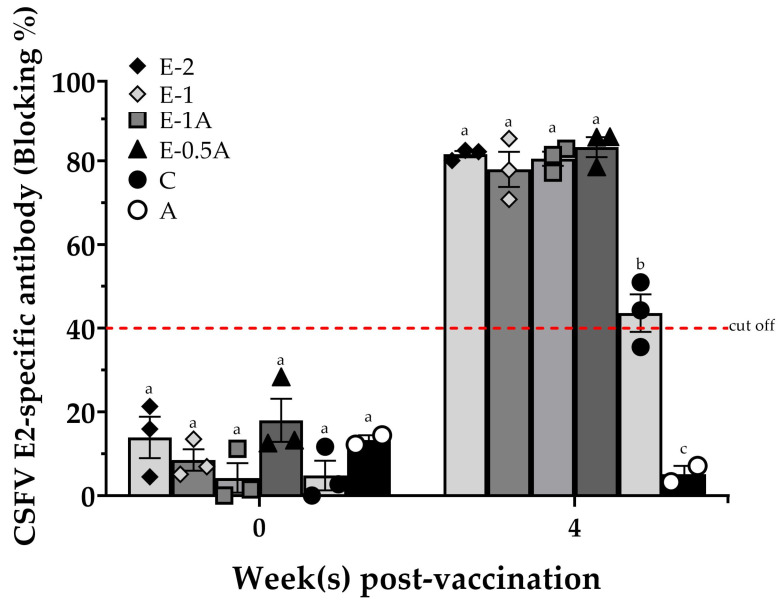
Anti-E2 antibodies 4 weeks post-vaccination in animal trial I. Sera samples from all the vaccinated pigs were collected and evaluated for E2-specific immune responses by IDEXX CSFV Ab test (blocking %). The results are expressed as a blocking percentage, and values ≥ 40% were considered positive. The data were presented as mean ± SEM. Statistical differences among groups were analyzed using one-way ANOVA followed by Duncan’s multiple range test. Different letters (^a, b, c^) indicate that the groups are significantly (*p* < 0.05) different from each other at the same time point.

In animal trial I, all the pigs displayed normal rectal temperatures (below 40.5 °C) and weight gains throughout the entire vaccination period. All the animals in Groups E-2, E-1, E-1A, and E-0.5A presented high levels of CSFV E2-specific antibodies (over 75% blocking) 4 weeks after primary vaccination. Those E2-specific antibody levels were significantly higher than those in Group C (slightly more than 40% blocking), which were vaccinated with a dose of a commercial vaccine adjusted to double dosage. In particular, the pigs in Group E-0.5A with a lower amount of E2 antigen (50 µg/dose) had robust immune responses (up to more than 80% blocking), which was no lower or even greater than that of the pigs in Groups E-1A and E-2 with 100 µg of E2 antigens in one or two doses. As expected, no positive E2-specific antibodies were observed in the negative control (Group A) (Figure 1).

On the other hand, the consistent response in E2-specific antibodies of the pigs in Group E-0.5A (83.3 ± 2.4% blocking) showed smaller individual differences than those in Group C (43.6 ± 4.5% blocking) (Table S2).

### 3.2. Assessment of the Clinical Signs and Mortality After CSFV Challenge in Animal Trial II

**Figure 2:**
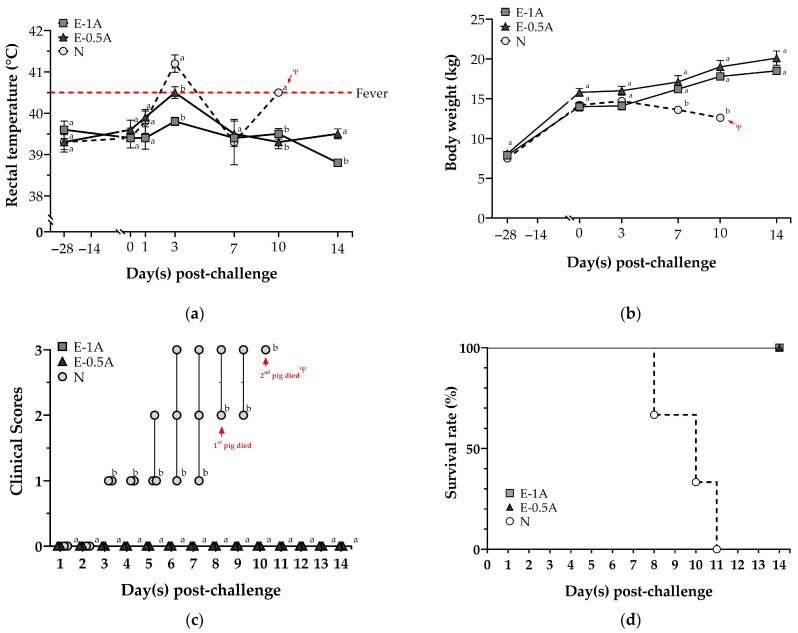
The clinical signs of the pigs were observed before and after CSFV challenge. Determination of (**a**) rectal temperature and (**b**) body weight in the pigs at −28, 0, 3, 7, 10, and 14 days post challenge (*dpc*). (**c**) The clinical scores of all the pigs were monitored daily and (**d**) survival curves of all the pigs during the CSFV challenge. The data were presented as mean ± SEM. Statistical differences among groups were analyzed using one-way ANOVA followed by Duncan’s multiple range test, while survival data were analyzed using the Mantel–Cox log-rank test. In addition, the non-parametric Mann–Whitney U test was used to analyze clinical scores, and data were expressed as the median with 95% confidence intervals. Different letters (^a, b^) indicate that the groups are significantly (*p* < 0.05) different from each other at the same time point. The Ψ symbol indicates that no data could be obtained due to a lack of pigs in Group N as a result of death at 11 *dpc*.

In animal trial II, the rectal temperature and body weight did not significantly differ among groups before CSFV challenge. The pigs in Group N without vaccination presented a fever at 3 days post-challenge (*dpc*) and hypothermia at 7 *dpc* (Figure 2a). After challenge with a highly virulent CSFV, the body weight of the pigs in Group N without vaccination kept decreasing from 3 *dpc* until death (Figure 2b). In contract, the pigs in the two vaccinated groups (Groups E-1A and E-0.5A) had no other clinical signs during the period of challenge study. Despite two of three pigs in Group E-0.5A having a slight fever at 3 *dpc* and returning to normal body temperatures within 48 h.

The health of the pigs without vaccination (Group N) began to be impaired at 3 *dpc* and started to show severe clinical signs including depression, anorexia, walking followed by shivering, skin hemorrhage, diarrhea, and neurological symptoms, which were the same as the classic infection symptoms of CSF. One of three pigs in Group N obtained the first severe score for clinical signs starting from 3 *dpc* and had the most severe CSF clinical symptoms (clinical score of 3) observed in all the pigs prior to mortality (Figure 2c). All non-vaccinated pigs (Group N) died before the end of the experiment (i.e., on days 8, 10, and 11 after challenge) (Figure 2d). Whereas all the vaccinated pigs in Groups E-1A and E-0.5A showed no clinical signs and held a clinical score of 0 after being challenged with a highly virulent CSFV strain and had a 100% survival rate.

### 3.3. Protection of the Pigs with E2-CpG Vaccination from Highly Virulent CSFV Challenge

**Figure 3:**
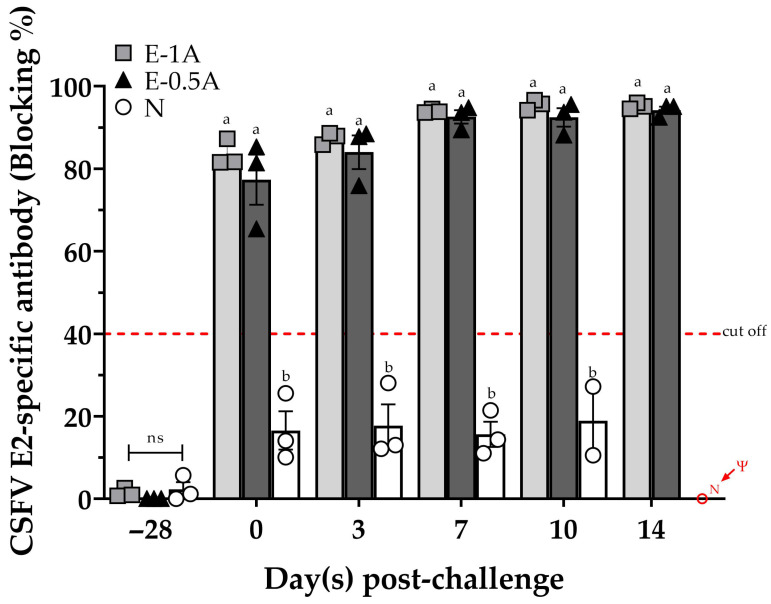
The response of E2-specific antibodies in pig sera was measured before and after CSFV challenge in animal trial II. Sera samples from all the pigs were collected and evaluated for E2-specific immune responses by IDEXX CSFV Ab test (blocking %). Results are expressed as blocking percentage, and values ≥ 40% were considered positive. The data were presented as mean ± SEM. Statistical differences among groups were analyzed using one-way ANOVA followed by Duncan’s multiple range test. Different letters (^a, b^) indicate that the groups are significantly (*p* < 0.05) different from each other at the same time point. The Ψ symbol indicates that no data could be obtained due to a lack of pigs in Group N as a result of death at 11 *dpc*.

Single-dose-immunized pigs in Groups E-1A and E-0.5A remained and continued to increase blocking rates of 77–96% in anti-E2 antibodies during the period of the 14-day CSFV challenge. Noticeably, pigs in Group E-0.5A with low E2 antigen showed almost equal levels of E2-specific antibodies as compared with those in Group E-1A with the high amount of E2 antigen. Expectedly, all the pigs in Group N had no detectable anti-E2 antibody before or after challenge (Figure 3).

Furthermore, the viral loads of CSFV from pigs in two vaccinated groups (Groups E-0.5A and E-1A) were completely cleared from serum, nasal, and saliva samples at 10 *dpc* prior to euthanasia. The saliva swabs of pigs in Group E-1A showed only transient, low-level viral RNA of CSFV at 7 *dpc*, and only one of three pigs in Group E-0.5A was also detected with viral loads of CSFV from sera and saliva swabs at 7 *dpc*. In stark contrast, pigs in the non-vaccinated Group N started to have detectable viral loads in serum and nasal samples as early as 3 *dpc* and their sera, nasal swabs, and saliva swabs had high levels of viral loads throughout at 7 *dpc* and 10 *dpc* till death (Table 2).

**Figure 4:**
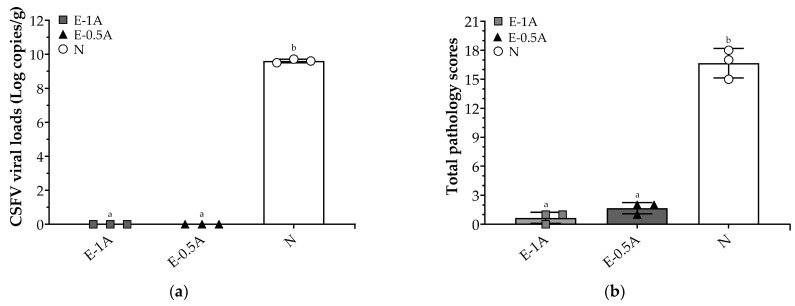
Detection of the viral loads in the spleen and total pathological scores evaluated in pigs vaccinated with the E2 subunit vaccine against CSFV. (**a**) Samples were taken from the spleens of pigs for quantification of CSFV viral loads by real-time RT-PCR. Data were expressed as mean ± SEM of log CSFV genomic copies/g. (**b**) Gross lesions and histopathological lesions were scored in pigs of three groups and summed into a total pathological score from six organs (cerebrum, spleen, kidney, lymph nodes, stomach, and ileocecal). The data were presented as mean ± SEM. Statistical differences among groups were analyzed using one-way ANOVA followed by Duncan’s multiple range test. Different letters (^a, b^) indicate that the groups are significantly (*p* < 0.05) different from each other at the same time point.

Post-challenge analysis at 14 *dpc* confirmed that spleen samples of pigs in two vaccinated groups were completely free of detectable CSFV RNA, even though those in Group E-0.5A were vaccinated with a lower antigen dose. Conversely, spleens in non-vaccinated pigs had high viral loads, reaching nearly 10^10^ copies/g (Figure 4a). These findings highlight that pigs with vaccination showed high levels of anti-E2 antibodies and the robust protective efficacy of both E2-CpG vaccine formulations in eliminating systemic and mucosal viral loads following challenge with a highly virulent CSFV strain.

### 3.4. Histopathological Analysis of Evaluated Vaccine Protection Against CSFV

At post-mortem examination, no gross lesions were observed in pigs from either vaccinated group (E-1A or E-0.5A). Histopathological lesions in vaccinated pigs were minimal, with Group E-1A showing only mild lymph node swelling and Group E-0.5A exhibiting slight lesions in the spleen, lymph nodes, and ileocecal region. In contrast, pigs in the non-vaccinated group (Group N) exhibited classical pathological lesions consistent with CSFV infection. These included encephalitis with perivascular cuffing, severe mesenteric vascular congestion in the spleen, petechial hemorrhages in the renal subcapsular region, necrotic debris, and hemorrhages in lymphoid follicles of lymph nodes, crypt abscesses to ulcers in the ileum, and diffuse hemorrhages throughout the gastrointestinal mucosa (Figure S2). Correspondingly, pathological scores remained low in vaccinated pigs, with average scores of 0.7 in Group E-1A and 1.7 in Group E-0.5A. In comparison, the non-vaccinated group reached a significantly high pathological score of 16.7 out of a maximum of 18 (Figure 4b).

Collectively, these findings confirm that a single administration with either E2-CpG vaccine formulations conferred complete protection against a highly virulent CSFV challenge, with the low-dosage E2 vaccine (Group E-0.5A) providing comparable protection to the higher-dosage group.

## 4. Discussion

Systematic vaccination is a highly effective strategy for reducing classical swine fever (CSF) epidemics and eradicating classical swine fever virus (CSFV) in the pig industry [7,15,16,17]. CSFV E2 subunit vaccines, which utilize viral surface antigens rather than the complete virus, enhance safety by eliminating the risk of residual virus replication associated with live attenuated vaccines [8,18,19,20,21,22] and do not interfere with the immune response in piglets with maternal antibodies [23,24]. Currently available commercial E2 subunit vaccines, such as Bayovac^®^ CSF and Porcilis^®^ Pesti, require booster administration to ensure complete protection against highly virulent CSFV strains [22,25,26,27]. Studies have shown that piglets immunized twice with CSFV E2 subunit vaccines at a three-week interval achieve an E2-specific antibody blocking rate of approximately 80% at 4–5 weeks post-primary vaccination when challenged with CSFV [28,29,30,31,32,33]. However, a two-dose commercial subunit vaccine (TWJ-E2^®^) administered to pigs resulted in CSFV viral loads of 10^10^–10^14^ copies/mL in blood, pharyngeal swabs, and tissues after CSFV challenge until euthanasia, with a 5–25% decline in the blocking rate of E2-specific antibodies between 3 and 9 days post-challenge [28]. This highlights the limitations of some subunit vaccines. In contrast, in this study, piglets vaccinated only once with the E-0.5A vaccine exhibited a high CSFV E2-specific antibody blocking rate of 83.3 ± 2.4% at four weeks post-vaccination in animal trial I (Figure 2). Previous studies have shown that CpG motifs primarily activate B cells and antigen-presenting cells through TLR9 engagement, thereby stimulating both innate and adaptive immune responses [34,35,36]. In this study, a porcine-specific CpG adjuvant designed by the corresponding authors (United States Patent No. US 10117929 B1) was incorporated into the vaccine formulation. According to the patent, this CpG adjuvant enhances the expression of co-stimulatory molecules such as CD80 and CD86 on peripheral blood mononuclear cells (PBMCs) and promotes Th1-type cytokine responses. These effects facilitate the activation of antigen-specific cytotoxic T cells and improve antibody quality, including avidity and neutralizing capacity. Therefore, pigs in both E2-CpG vaccine groups remained healthy and exhibited no severe clinical signs or pathological lesions (Figure S2) during the 14-day CSFV challenge period in animal trial II. The vaccines successfully cleared the CSFV viral loads in pigs with no detectable viral shedding in sera, nasal swabs, saliva swabs (Table 2), or spleen samples (Figure 4a). These findings suggest that a single dose of the E2-CpG vaccine is highly effective in preventing viral replication, clearing highly virulent CSFV, and probably inducing a Th1-biased immune profile, providing robust clinical protection against CSF at four weeks post-vaccination.

However, a single dose is used in a subunit vaccine, and a higher dosage is required to achieve the protective effect. Multiple immunizations or higher antigen amounts increase vaccine costs and impose an economic burden on pig farms. Previous studies have shown that animals were immunized with a high dosage of E2 antigen without booster vaccination, ranging from 100 µg to 300 µg [37,38,39,40]. Therefore, researchers have been exploring whether a single-dose lower-dose vaccine can provide comprehensive protection against CSFV while maintaining cost-effectiveness for clinical application. As Madera et al. (2016 and 2018) [32,41] reported, a single dose of their study vaccine protected pigs from CSFV challenge. E2-specific antibody levels increased in vaccinated pigs receiving 50 µg and 75 µg vaccine dosages within 2 to 3 weeks post-vaccination but gradually declined during the 15-day CSFV challenge period from 9 days post-challenge (*dpc*) to 15 *dpc* [41]. Notably, the amount of E2 protein used in their vaccine exceeded the dosage in the Bayovac^®^ CSFV E2 subunit vaccine (more than 32 µg) [32], but required a booster vaccination (total amount up to 60 µg).

Hence, we evaluated whether the study vaccine could achieve protective efficacy against CSFV with a single, lower-dosage administration in this study. For this purpose, the Bayovac^®^ CSFV E2 subunit vaccine was adjusted to a 64 µg dosage for comparison. Notably, the pigs in Group E-0.5A (50 µg) demonstrated an approximately 50% higher E2-specific antibody blocking rate than those in Group C 4 weeks post-vaccination, comparable to the pigs in Groups E-1A (100 µg) and E-2 (200 µg) and even exceeding those groups (Figure 2). In addition, the Bayovac^®^ CSFV E2 subunit vaccine (Group C-2) was administered twice at two-week intervals in our initial studies, following product guidelines. The pigs in Group C-2 exhibited higher variance in E2-specific antibody blocking rates (22.7 ± 10.0% and 64.3 ± 11.9%) compared with those in Group E-2 (26.3 ± 9.0% and 71.4 ± 3.2%) at 2 and 4 weeks post-vaccination, respectively. As previous reports indicate, naïve young pigs vaccinated with the Bayovac^®^ CSFV E2 vaccine showed significant individual variation in E2 antibody titers at 3 weeks post-vaccination, which did not stabilize until 6 weeks after booster administration in field farm applications [26]. Interestingly, the pigs in Group E-0.5A demonstrated less variation in individual E2-specific antibody responses, exhibiting greater consistency compared with those receiving booster (Group C-2) or double-dosage (Group C) vaccination in the initial study or animal trial I. Variance values in Groups C and C-2 were 3.6-fold and 16.7-fold higher, respectively, compared with Group E-0.5A (Table S2). Furthermore, the pigs in the E-0.5A group showed high levels of E2-specific antibodies 4 weeks after vaccination, and the antibody levels did not decline in the serum during the 14-day CSFV challenge, with the response remaining persistently high. However, this study focused primarily on humoral immune responses and did not include detailed analysis of cellular immunity. It also lacks information on the duration of protective immunity after the short-term post-vaccination (4 weeks). It will be essential to determine the practical duration of immunity with long-term follow-up studies and guide field vaccination schedules, such as evaluating antibody persistence and protective efficacy at 6 months or 1 year post-vaccination. Future studies hope to clarify a detailed immunoprofiling of cytokine expression and T cell subsets to further elucidate the cellular mechanisms underlying the potent protection observed with the E2-CpG formulation. Nevertheless, the findings of this study demonstrate that the E-0.5A vaccine produces an antibody response with a lower dose and a single vaccination, which effectively protects against CSFV. This approach reduces vaccination costs and labor, making it a more practical solution for pig farm operations.

The development of subunit vaccines is a key strategy for addressing the inability of live attenuated vaccines to differentiate between infected and vaccinated animals (DIVA) [16,17,42,43]. Live attenuated vaccines pose challenges in serological diagnostics because they induce antibodies against both structural (E2 and E^rns^) and nonstructural (NS3) proteins of CSFV, making it difficult to distinguish between vaccinated and naturally infected animals [20,44,45,46]. In contrast, animals vaccinated with the CSFV E2 subunit vaccine produce only CSFV E2-specific antibodies but not E^rns^- or NS3-specific antibodies, allowing their use in DIVA strategies [28,29,30,38,47,48,49,50,51,52]. Taiwan has not reported any cases of CSF since 2006 and was officially recognized as free of the disease by the World Organisation for Animal Health (WOAH) at the 92nd General Session in May 2025. Notably, in 2014, accidental administration of a live attenuated vaccine (LAV-LOM strain) to sows in Jeju Island, South Korea, resulted in an outbreak of CSF in previously uninfected regions [53]. Hence, CSF epidemics have reemerged in Asian countries neighboring Taiwan, including Japan [54] and South Korea [55]. As Bouma et al. (1999) suggested, a country preparing to cease CSFV vaccination and pursue virus eradication must investigate the suitability of E2 marker vaccines for use in outbreak control programs [19]. Despite their broad protection and effectiveness in controlling CSF epidemics in recent years [8,17,20,55,56], live attenuated vaccines must be carefully managed. The implementation of vaccination strategies in CSF-free areas is critical to mitigating the risk of global CSF re-emergence due to improper live attenuated vaccine application [57,58,59,60]. Such as incorrect dosage or failure to establish optimal herd immunity, can lead to serious consequences, including the emergence of escape variants of CSFV strains [58,61] and the resurgence of the disease [21,54,62]. The production of DIVA-compatible vaccines is essential for eradicating or controlling CSF, as subunit vaccines combined with appropriate diagnostic reagents enable relevant authorities in endemic regions to implement successful prevention and control strategies [16,42].

In this study, a DIVA differential diagnostic kit currently under development was used to analyze serum samples. Sows vaccinated with live attenuated vaccines tested positive for E^rns^ antibodies, while sera from pigs in Groups E2-CpG, C, and SPF pigs showed negative responses (Figure S3), confirming the potential utility of the subunit vaccine in DIVA strategies. Nevertheless, the E2-CpG vaccine was designed for the genotype 2.1 strain that has become predominant in recent outbreaks across East and Southeast Asia. This study evaluated the efficacy of the vaccine to protect against the highly virulent ALD strain (genotype 1.1) of CSFV, suggesting the potential for cross-protection against isolates of other genotypes. Future investigations will be essential to confirm the cross-protective potential of the E2-CpG vaccine and its broader regional applicability. Moving forward, a CSFV E2 subunit vaccine formulated with a porcine CpG adjuvant and a commercial adjuvant that successfully protects pigs against CSFV can be deployed in disease-endemic regions alongside diagnostic tools, reinforcing stringent measures to prevent multiple subgenotype viral invasions and enhance CSF control strategies.

## 5. Conclusions

In the present study, pigs in Group E-0.5A showed the highest levels of CSFV E2-specific antibodies in comparison to the other groups 4 weeks after vaccination. After CSFV challenge, pigs with the lower dosage and single-dose vaccines (E-0.5A) presented a similar level of E2-specific antibodies as produced in the high-dosage vaccine (E-1A), while the viremia completely disappeared before euthanasia. In conclusion, a lower dosage of antigen and a single dose of a new formula of the E2-CpG vaccine can effectively stimulate immunity against a highly virulent CSFV (CSFV ALD) strain.

## Figures and Tables

**Table 1:** Vaccination treatments for two animal trials.

Group * (n)	Vaccination Treatments	
	CSFV E2 Protein (µg/Dose)	CpG Adjuvant (µg/Dose)
**Animal Trial I**		
E-2 (3)	200	0
E-1 (3)	100	0
E-1A (3)	100	100
E-0.5A (3)	50	100
C (3)	>64	0
A (2)	0	100
**Animal Trial II**		
E-1A (3)	100	100
E-0.5A (3)	50	100
N (3)	0	0

* All pigs were primed intramuscularly at 4 weeks of age; only groups E-2 and C received a booster administration with the same dose after 2 weeks. C: The package insert states that the Bayovac^®^ CSF-E2 vaccine includes a minimum of a 32 μg/dose of CSFV E2 glycoprotein in 2 mL, which was adjusted to a single dose with 64 μg/dose of CSFV E2 glycoprotein in 2 mL; A: It represented CpG adjuvant; N: The piglets were injected with saline (2 mL/dose).

**Table 2:** Pigs vaccinated with E2 subunit vaccine did not develop viremia, fecal shedding, and nasal shedding after CSFV challenge.

Group (*n*) *	CSFV Viral Loads in Serum, Nasal, and Saliva Samples Before and Post-Challenge (*dpc*)					
	Ct			Log Copies/mL		
	E-1A (3)	E-0.5A (3)	N (3)	E-1A (3)	E-0.5A (3)	N (3)
	**Sera**					
−28 *dpc*	(−)	(−)	(−)	0 ^a^	0 ^a^	0 ^a^
0 *dpc*	(−)	(−)	(−)	0 ^a^	0 ^a^	0 ^a^
3 *dpc*	(−)	32.2 ± 0.8	30.3 ± 0.8	0 ^a^	3.2 ± 1.2 ^ab^	5.4 ± 0.3 ^b^
7 *dpc*	(−)	32.8 **^⁘^**	18.0 ± 0.3	0 ^a^	1.5 ± 1.2 ^a^	9.1 ± 0.1 ^b^
10 *dpc*	(−)	(−)	17.1 ± 0.8	0 ^a^	0 ^a^	9.4 ± 0.3 ^b^
14 *dpc*	(−)	(−)	N/A	0 ^a^	0 ^a^	N/A
	**Nasal**					
−28 *dpc*	(−)	(−)	(−)	0 ^a^	0 ^a^	0 ^a^
0 *dpc*	(−)	(−)	(−)	0 ^a^	0 ^a^	0 ^a^
3 *dpc*	(−)	(−)	32.0 **^⁘^**	0 ^a^	0 ^a^	1.7 ± 1.3 ^a^
7 *dpc*	(−)	(−)	27.2 ± 1.1	0 ^a^	0 ^a^	6.5 ± 0.3 ^b^
10 *dpc*	(−)	(−)	24.7 ± 0.0	0 ^a^	0 ^a^	7.2 ± 0.0 ^b^
14 *dpc*	(−)	(−)	N/A	0 ^a^	0 ^a^	N/A
	**Saliva**					
−28 *dpc*	(−)	(−)	(−)	0 ^a^	0 ^a^	0 ^a^
0 *dpc*	(−)	(−)	(−)	0 ^a^	0 ^a^	0 ^a^
3 *dpc*	(−)	(−)	(−)	0 ^a^	0 ^a^	0 ^a^
7 *dpc*	35.3 ± 0.7	37.6 **^⁘^**	27.7 ± 1.0	3.4 ± 0.2 ^ab^	1.3 ± 1.3 ^a^	5.8 ± 0.3 ^b^
10 *dpc*	(−)	(−)	26.2 ± 0.7	0 ^a^	0 ^a^	6.3 ± 0.2 ^b^
14 *dpc*	(−)	(−)	N/A	0 ^a^	0 ^a^	N/A

* All pigs were primary vaccinated intramuscularly at 4 weeks of age and were immunized with E-1A, E-0.5A, and N (saline), respectively, on −28 days post-challenge (*dpc*). All pigs were challenged with CSFV four weeks after vaccination and sacrificed on day 14 post-challenge. Data are presented as Ct values, transformed into mean ± SEM of Log copies/mL. Statistical differences among groups were analyzed using one-way ANOVA followed by Duncan’s multiple range test. Different letters (^a, b^) indicate that the groups are significantly (*p* < 0.05) different from each other at the same time point. (−) represents an undetectable Ct value by qPCR analysis; N/A shows that data is not available due to lack of animals as a result of death from CSFV at 8–10 *dpc*. ⁘ Of the three pigs examined, only one yielded a detectable Ct value, thus only showing Ct value data for one of those; for statistical analysis, the two undetectable samples were represented as 0 log copies/mL.

## Data Availability

The raw data supporting the conclusions of this article will be made available by the authors on request.

## Supplementary Materials

The following supporting information can be downloaded at: https://www.mdpi.com/article/10.3390/vaccines13101072/s1, Figure S1: SDS-PAGE and Western-blot of CSFV E2 antigen; Figure S2: Gross and histopathological lesions of the vaccinated pigs after CSFV challenge; Figure S3: Application and evaluation of CSFV E^rns^ antigen in an ELISA diagnostic assay; Table S1: The amino acid sequence of CSFV E2 antigen expressed by the baculovirus system; Table S2: CSFV E2-specific antibody response before and after vaccination by IDEXX CSFV Ab test; uncropped Western blots.

## Abbreviations

The following abbreviations are used in this manuscript:

CSF	Classical swine fever
CSFV	Classical swine fever virus
SPF	Specific-pathogen-free
WOAH	World Organisation for Animal Health
DIVA	Differentiate between infected and vaccinated animals
AHRI	Veterinary Research Institute, Ministry of Agriculture (Miaoli, Taiwan)
*dpc*	Days post-challenge
mAbs	Monoclonal antibodies
ABSLs	Animal Biosafety Levels
ELISA	Enzyme-linked immunosorbent assay
H&E	Hematoxylin and eosin
SDS-PAGE	Sodium dodecylsulfate-polyacrylamide gel electrophoresis
β-ME	β-mercaptoethanol
FAID_50_	50% Fluorescent antibody infective dose
IACUC	Institutional Animal Care and Use Committee
