# Supplementary material for: Efficacy Evaluation of an E2 Subunit Vaccine Against Highly Virulent Classical Swine Fever Virus Strain
Source: Vaccines (Basel). 2025 Oct 20;13(10):1072. doi: 10.3390/vaccines13101072 (PMC12568282; doi:10.3390/vaccines13101072)

Uncropped Western blots of Figure S1a,b

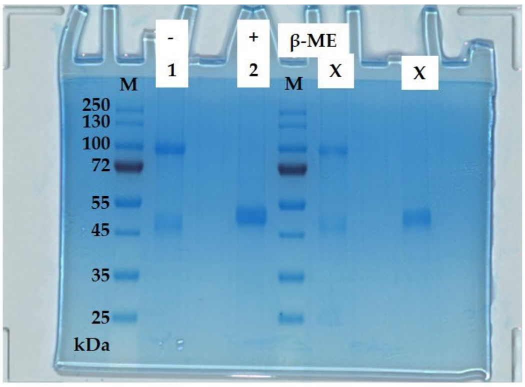

(a)

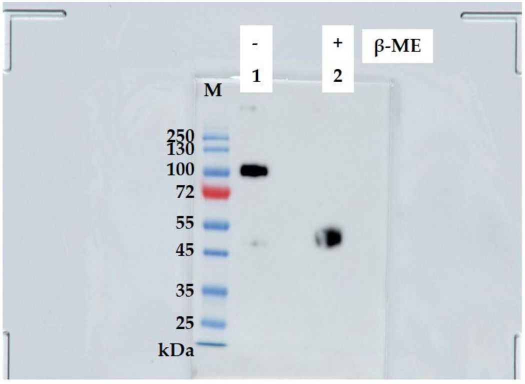

(b)

Uncropped Western blots of Figure S1a

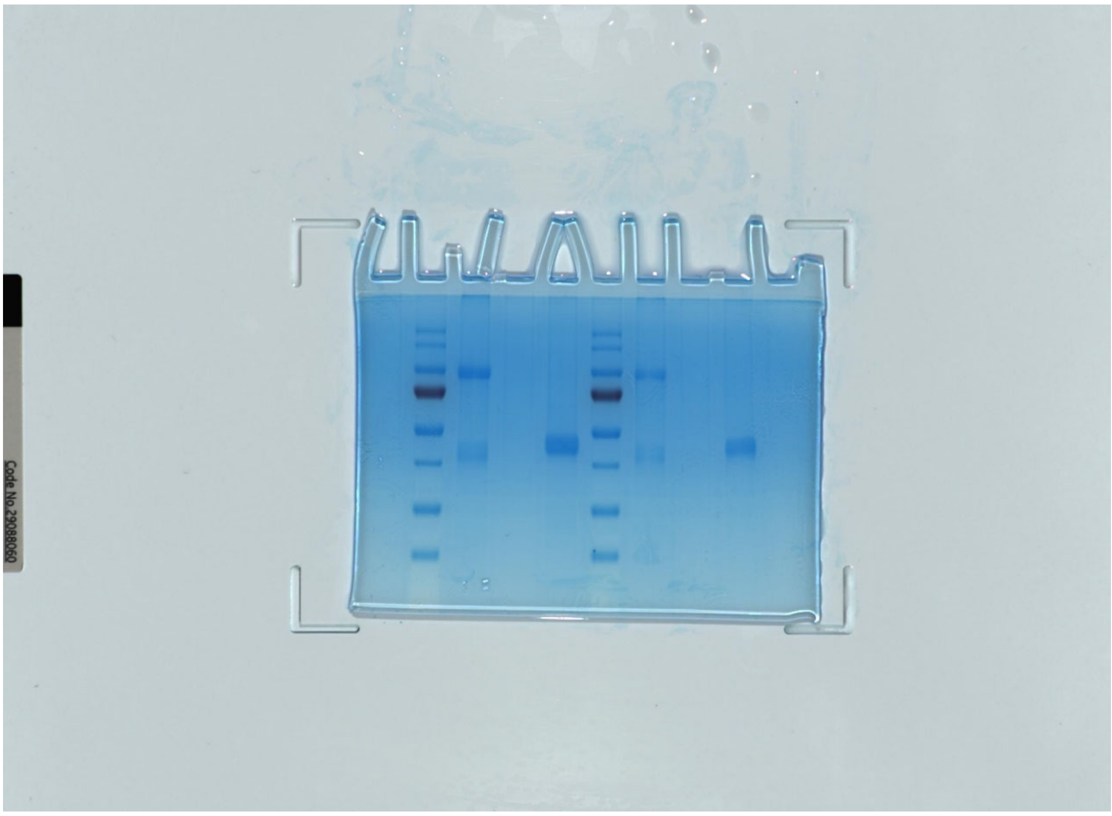

Uncropped Western blots of Figure S1b

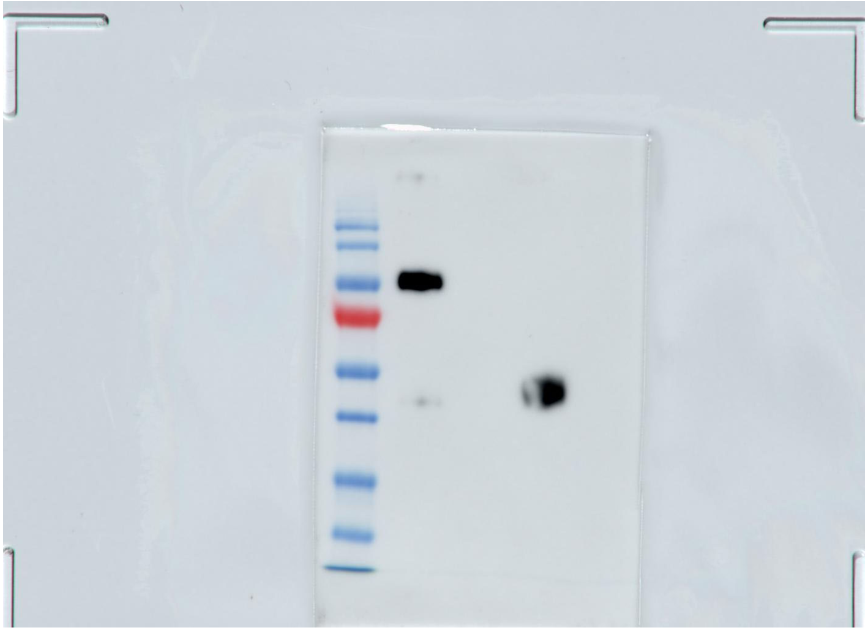

Uncropped Western blots of Figure S3a,b

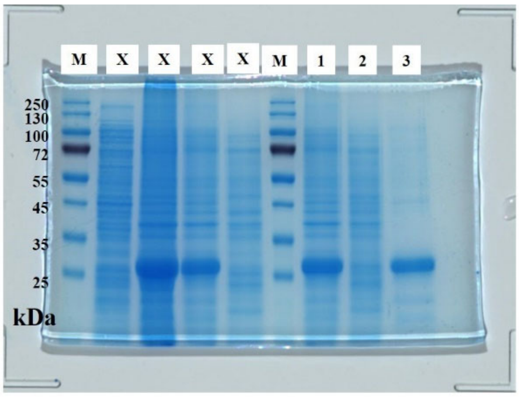

(a)

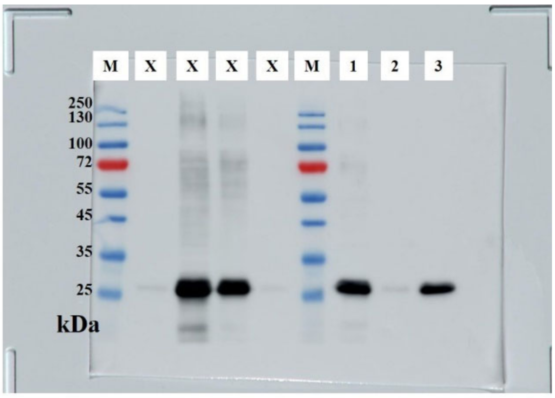

(b)

Uncropped Western blots of Figure S3a

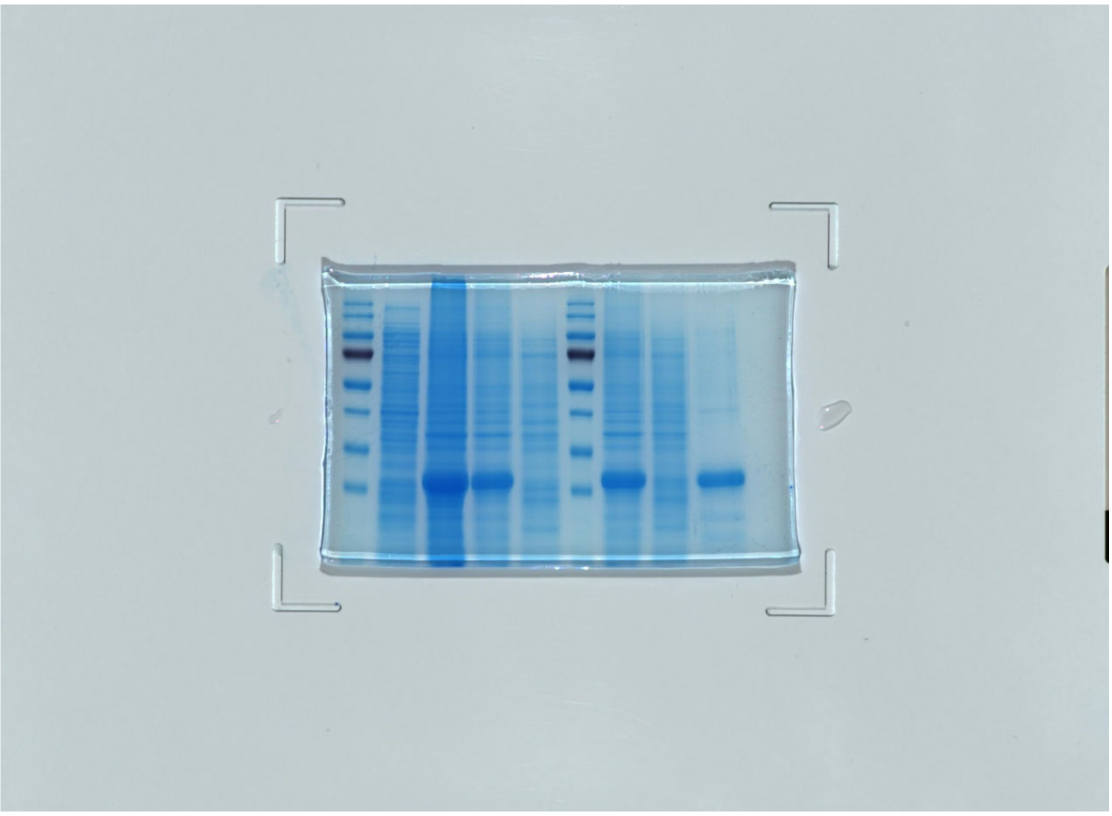

Uncropped Western blots of Figure S3b

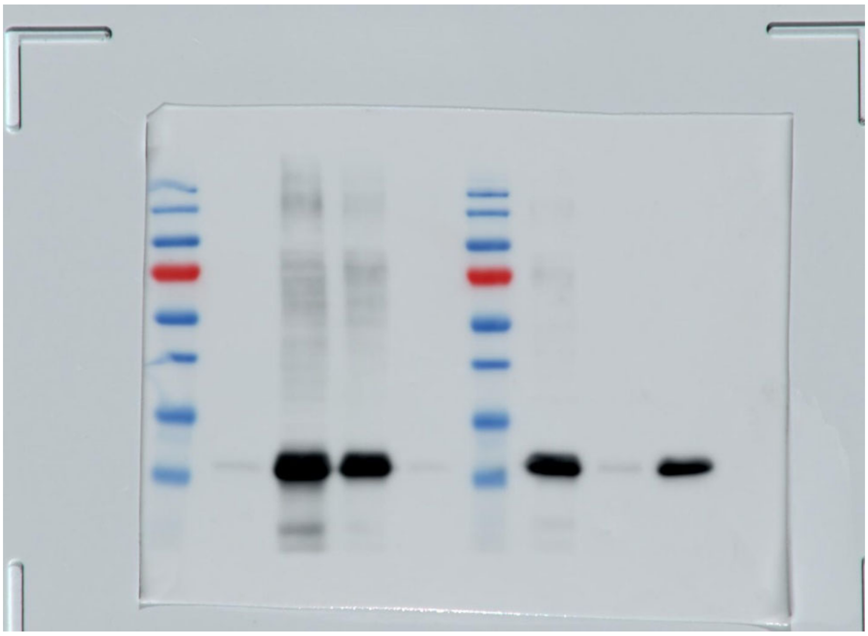

Supplement: Supplementary file 1 [file vaccines-13-01072-s001.zip › uncropped Western blots.pdf]
