# Supplementary material for: Efficacy Evaluation of an E2 Subunit Vaccine Against Highly Virulent Classical Swine Fever Virus Strain
Source: Vaccines (Basel). 2025 Oct 20;13(10):1072. doi: 10.3390/vaccines13101072 (PMC12568282; doi:10.3390/vaccines13101072)
Supplement: Supplementary file 1 [file vaccines-13-01072-s001.zip › supplementary Figures and tables.pdf]

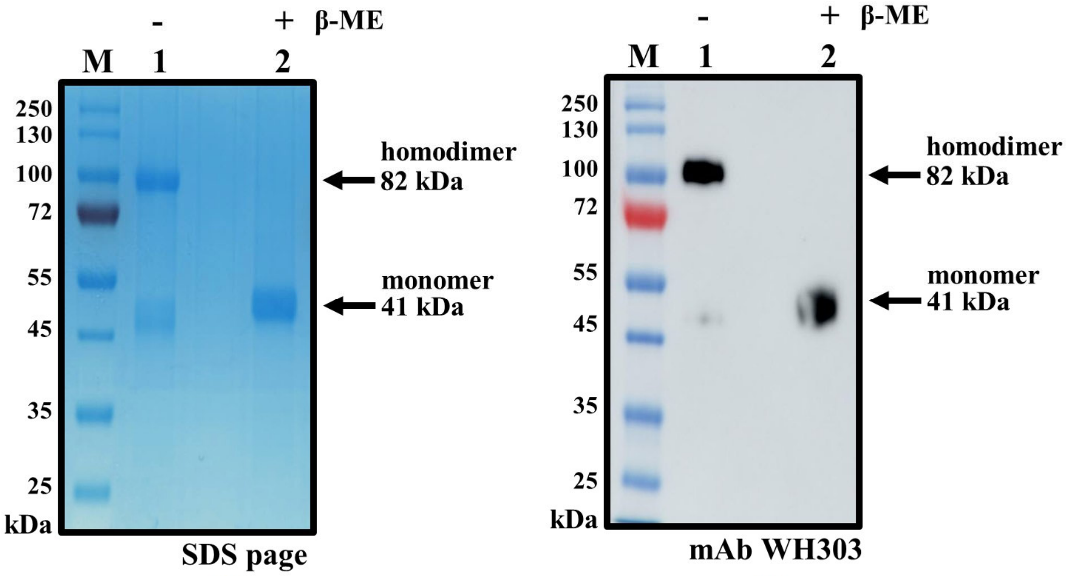

(a) (b)

Figure S1. SDS-PAGE and Western-blot of CSFV E2 antigen. (a) SDS-PAGE analysis with or without E2 antigen (1 μg) reducing reagent β-ME (Lanel and Lane2, respectively).(b) Western-blot of E2 antigen. E2-specific mAb WH303 was used for the western-blot.

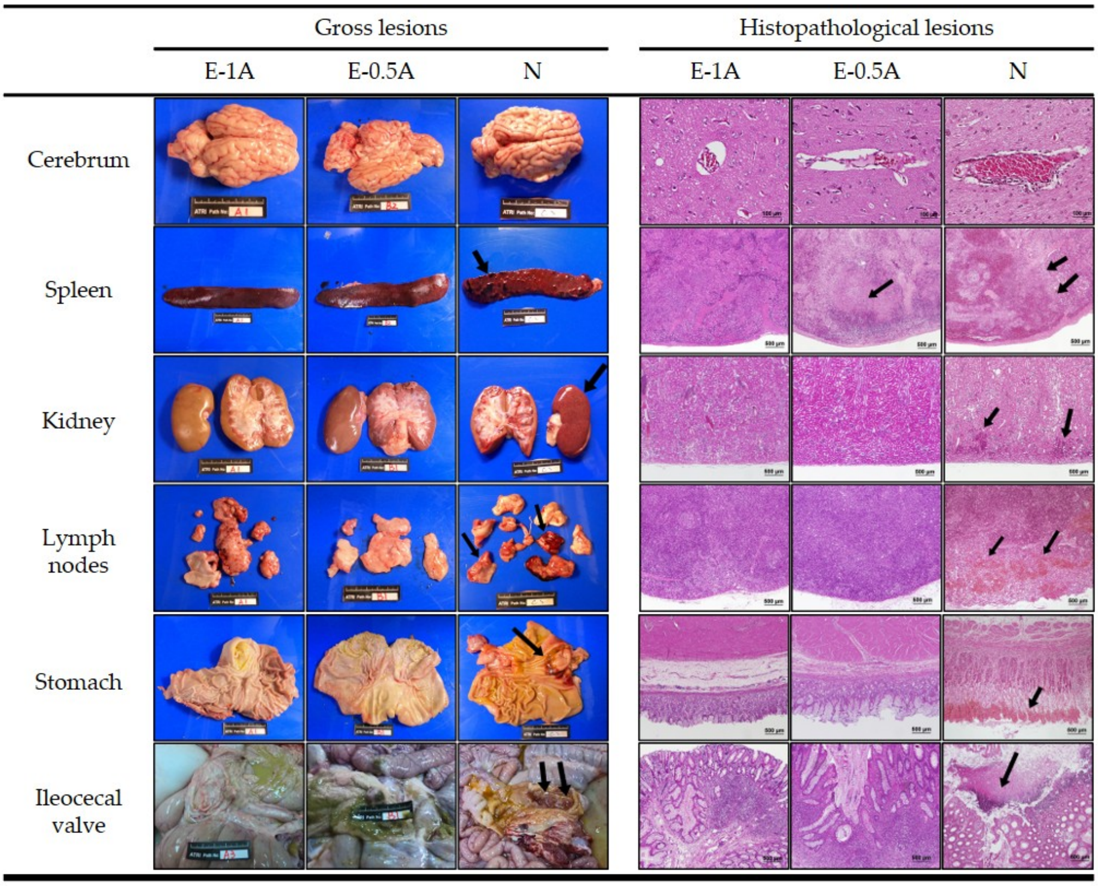

Figure S2. Gross and histopathological lesions of the vaccinated pigs after CSFV challenge. After a 14-day CSFV challenge, all lymphoid tissues and the meant organs were collected. Gross and histopathological

lesion analysis on major tissues (cerebrum, spleen, kidney, lymph nodes, stomach, and ileocecal valve) were carried out. In order to examine the major tissues by histopathology, they were sectioned before being stained with hematoxylin and eosin. The slides were examined under an optical microscope(Olympus BX51, Taiwan) at 40 to 600× magnification.

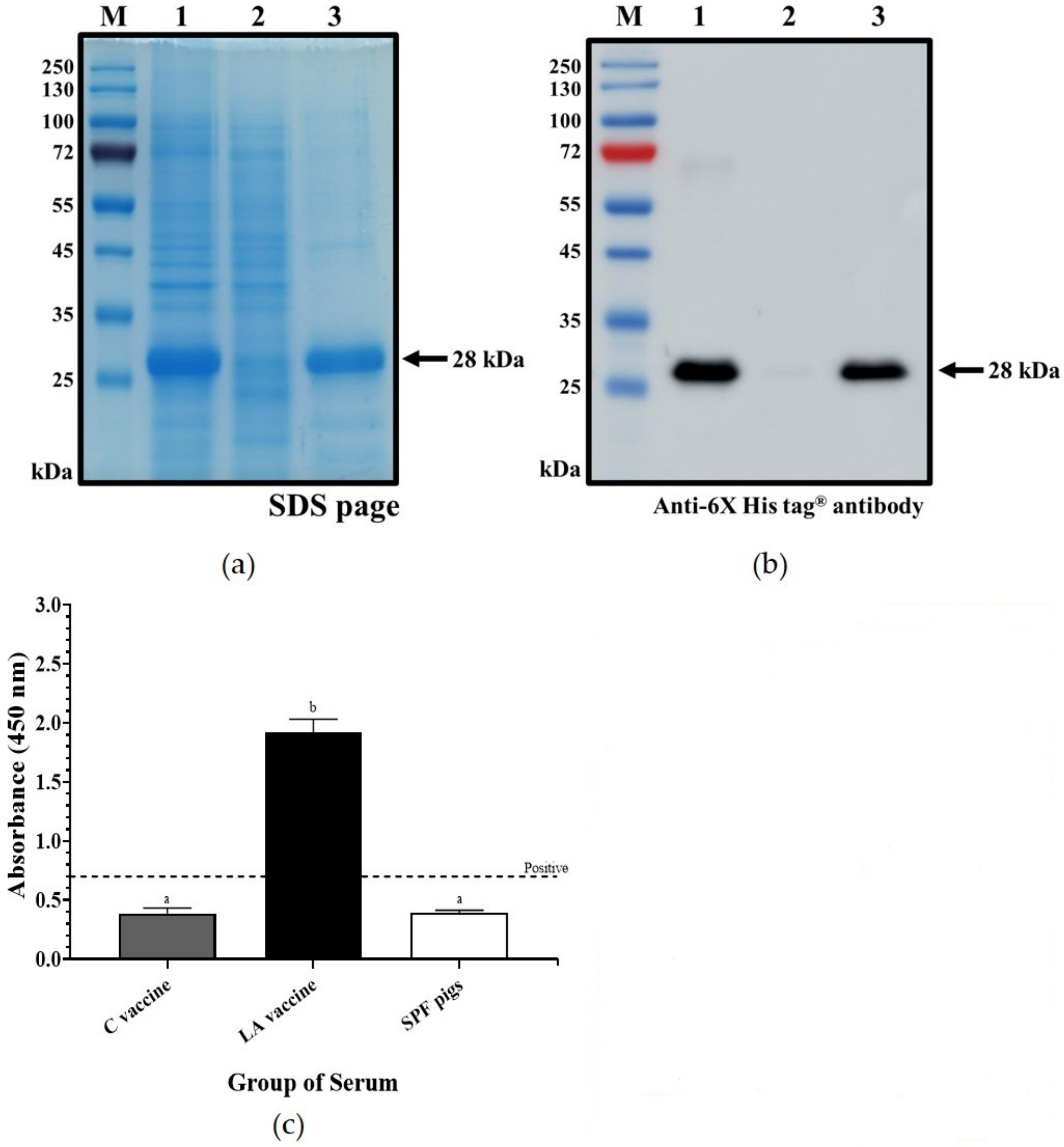

Figure S3. Application and evaluation of CSFV E^{ms} antigen in an ELISA diagnostic assay. SDS-PAGE (a) and Western blot (b) analysis of CSFV E^{ms} antigen purification and analyzation. (Lane 1: supernatant after cell lysis; Lane 2: flow-through fraction following affinity chromatography; Lane 3: purified E^{ms} antigen) For Western blot analysis, the purified E^{ms} antigen fused with a 6×His tag ab18184; Abcam, Cambridge, MA, USA) was detected using an anti-His antibody. A homemade ELISA was developed using E^{ms}-coated plates to evaluate serum samples, with optical density (OD) values measured at 450 nm (C). For assay validation, the Positive Control (PC)was required to have an $OD_{450} \geq 0.7$, and the Negative Control (NC)s 0.5. The E2-CpG group consisted of sera from this study's vaccines. The C vaccine group consisted of sera from SPF pigs immunized with the Bayovac® CSF-E2 subunit vaccine. The LA vaccine group included

sera from pigs vaccinated with a commercial live attenuated CSFV vaccine. The SPF group represented sera from non-vaccinated, CSFV-antibody-negative SPF pigs.

Table S1. The amino acid sequence of CSFV E2 antigen expressed by the baculovirus system.

SPdTM (E2)	Sequence
Amino acid	MKVLRGQIVQGIIWLLLVTGAQGRLSCKEDHRYAISSTNEIGPL<br>GAEGLTTTWKEYNHGLQLDDGTVRAICIAGSFRVTALNVVSR<br>RYLASLHKRALPTSVIFELLFDGTSPAIEEMGDDFGFGLCPFDT<br>TPVVKGKYNTTLLNGSAFYLVCPIGWTGVIECTAVSPTTLRTE<br>VVKTFKREKPFPHRADCVTTIVEKEDLFHCKLGGNWTCVKGN<br>PVTYTGGQVKQCRWCGFDFKEPDGLPHYPIGKCILANETGYRI<br>VDSTDCNRDGVVVSTEGEHECLIGNTTVKVYALDGRLAPMPC<br>RPKEIISSAGPVRKTSCTFNYTKTLRNKYYEPRDSYFQQYMLKG<br>EYQYWFDLDVTGHHADYFAEGHHHHHH

Table S2. CSFV E2-specific antibody response before and after vaccination by IDEXX CSFV Ab test.

Groups (*n*)*	E2-specific antibodies (blocking%)** before (0 *wpv*) vaccination, and after 2 and 4 weeks of vaccination (*wpv*)			
	0 *wpv*	2 *wpv*	4 *wpv*	Variance***
**Initial study**				
E-2 (4)	2.6± 1.3[a]	26.3± 9.0[a]	71.4± 3.2[a]	61.0
C-2 (4)	0.0± 0.0[a]	22.7± 10.0[a]	64.3± 10.3[a]	283.3
**Animal Trail I**				
E-2 (3)	13.9± 5.0[a]		81.6± 0.8[a]	1.7
E-1 (3)	8.5± 2.6[a]		78.0± 4.2[a]	52.6
E-1A (3)	4.2± 3.5[a]		80.5± 1.7[a]	8.7
E-0.5A (3)	18.0± 5.2[a]		83.3± 2.4[a]	17.0
C (3)	4.8± 3.5[a]		43.6± 4.5[b]	60.4
A (2)	13.3± 1.1[a]		5.2± 2.0[c]	7.6

*All pigs were primarily vaccinated intramuscularly at 4 weeks of age; only Group E-2 and C-2 received a booster administration with the same dose after 2 weeks. Groups C-2 and C were a commercial vaccine (Bayovac® CSF-E2 vaccine, Bayer Taiwan Co., Ltd.) including the CSFV E2 glycoprotein at a minimum of 32 μg/dose in 2 mL, according to the package insert. C: It was adjusted to a single dose with 64 μg/dose of CSFV E2 glycoprotein in 2 mL; A: It represented CpG adjuvant.

[**]Results are expressed as blocking percentage, and values ≥ 40% were considered positive.

[***] Variance was calculated by $v_{ar}(x) = E[(x - \mu)^2]$. The data indicates the individual difference in the pigs of E2-specific antibodies in each group of pigs four weeks post-vaccination. The larger the value, the greater the degree of dispersion from the mean.
